# Extreme Dysbiosis of the Microbiome in Critical Illness

**DOI:** 10.1128/mSphere.00199-16

**Published:** 2016-08-31

**Authors:** Daniel McDonald, Gail Ackermann, Ludmila Khailova, Christine Baird, Daren Heyland, Rosemary Kozar, Margot Lemieux, Karrie Derenski, Judy King, Christine Vis-Kampen, Rob Knight, Paul E. Wischmeyer

**Affiliations:** a Department of Pediatrics, University of California San Diego, La Jolla, California, USA; b Department of Anesthesiology and Pediatrics (Nutrition Section), University of Colorado Denver, Aurora, Colorado, USA; c Department of Critical Care Medicine, Queen’s University and Clinical Evaluation Research Unit, Kingston General Hospital, Kingston, Ontario, Canada; d Shock Trauma Center, University of Maryland, University of Maryland Medical Center, Baltimore, Maryland, USA; e Department of Pharmacy, Cox Health, Springfield, Missouri, USA; f Critical Care Department, Southlake Regional Health Centre, Newmarket, Ontario, Canada; DOE Joint Genome Institute

**Keywords:** 16S RNA, critical care, fecal organisms, human, microbial source tracking

## Abstract

Critical illness is hypothesized to associate with loss of “health-promoting” commensal microbes and overgrowth of pathogenic bacteria (dysbiosis). This dysbiosis is believed to increase susceptibility to nosocomial infections, sepsis, and organ failure. A trial with prospective monitoring of the intensive care unit (ICU) patient microbiome using culture-independent techniques to confirm and characterize this dysbiosis is thus urgently needed. Characterizing ICU patient microbiome changes may provide first steps toward the development of diagnostic and therapeutic interventions using microbiome signatures. To characterize the ICU patient microbiome, we collected fecal, oral, and skin samples from 115 mixed ICU patients across four centers in the United States and Canada. Samples were collected at two time points: within 48 h of ICU admission, and at ICU discharge or on ICU day 10. Sample collection and processing were performed according to Earth Microbiome Project protocols. We applied SourceTracker to assess the source composition of ICU patient samples by using Qiita, including samples from the American Gut Project (AGP), mammalian corpse decomposition samples, childhood (Global Gut study), and house surfaces. Our results demonstrate that critical illness leads to significant and rapid dysbiosis. Many taxons significantly depleted from ICU patients versus AGP healthy controls are key “health-promoting” organisms, and overgrowth of known pathogens was frequent. Source compositions of ICU patient samples are largely uncharacteristic of the expected community type. Between time points and within a patient, the source composition changed dramatically. Our initial results show great promise for microbiome signatures as diagnostic markers and guides to therapeutic interventions in the ICU to repopulate the normal, “health-promoting” microbiome and thereby improve patient outcomes.

**IMPORTANCE** Critical illness may be associated with the loss of normal, “health promoting” bacteria, allowing overgrowth of disease-promoting pathogenic bacteria (dysbiosis), which, in turn, makes patients susceptible to hospital-acquired infections, sepsis, and organ failure. This has significant world health implications, because sepsis is becoming a leading cause of death worldwide, and hospital-acquired infections contribute to significant illness and increased costs. Thus, a trial that monitors the ICU patient microbiome to confirm and characterize this hypothesis is urgently needed. Our study analyzed the microbiomes of 115 critically ill subjects and demonstrated rapid dysbiosis from unexpected environmental sources after ICU admission. These data may provide the first steps toward defining targeted therapies that correct potentially “illness-promoting” dysbiosis with probiotics or with targeted, multimicrobe synthetic “stool pills” that restore a healthy microbiome in the ICU setting to improve patient outcomes.

**Podcast**: A podcast concerning this article is available.

## Abstract (precis)

Critical illness may be associated with the loss of normal, “health promoting” bacteria, allowing overgrowth of disease-promoting pathogenic bacteria (dysbiosis), which, in turn, makes patients susceptible to hospital-acquired infections, sepsis, and organ failure. This has significant world health implications, because sepsis is becoming a leading cause of death worldwide, and hospital-acquired infections contribute to significant illness and increased costs. Thus, a trial that monitors the ICU patient microbiome to confirm and characterize this hypothesis is urgently needed. Our study analyzed the microbiomes of 115 critically ill subjects and demonstrated rapid dysbiosis from unexpected environmental sources after ICU admission. These data may provide the first steps toward defining targeted therapies that correct potentially “illness-promoting” dysbiosis with probiotics or with targeted, multimicrobe synthetic “stool pills” that restore a healthy microbiome in the ICU setting to improve patient outcomes.

## OBSERVATION

What constitutes a healthy microbiome is poorly understood, as “healthy” likely depends on host genetics, environment, nutrition, age, and lifestyle (1). Although what is “healthy” is not yet well defined, identifying large-scale dysbiosis of the microbiome is increasingly feasible (2, 3). Critical illness leads to the admission of >5.7 million patients annually to intensive care units (ICUs) in the United States for intensive or invasive monitoring. These admissions consume ~20% of U.S. hospital costs, with death rates from critical illness increasing at a rate greater than that of any other common cause of death worldwide (4). Small, culture-based studies suggest that critical illness may be associated with loss of commensal microbes and overgrowth of potentially pathogenic and inflammatory bacteria. This dysbiosis is believed to lead to high susceptibility to hospital-acquired infections (HAIs), sepsis, and multiorgan dysfunction syndrome (MODS) (5–7); sepsis alone is becoming a leading cause of death worldwide (5), while HAIs in general contribute significantly to patient morbidity and increased costs. Pilot (<15 patients) microbiome studies of ICU patients have been undertaken (8, 9), and the results of these explorations suggest the urgent need for larger prospective studies that monitor the microbiome of critical-care patients by using culture-independent techniques to test the long-held dysbiosis hypothesis (10).

To address this, we set out to characterize the microbiomes of 115 critically ill (ICU) patients at two time points. The novelties of this study relative to prior investigations are that we applied a probabilistic framework, SourceTracker (11), to assess whether the microbial communities appear to source from expected compositions; assessed differential taxonomic abundances with ANCOM (12) relative to the American Gut Project (AGP); and performed a co-occurrence analysis of the ICU patient samples by using SparCC (13). The results of this study show that, regardless of the reason for admission, the microbiome of many critical-care patients is drawn from unexpected sources (i.e., fecal not resembling fecal), the individuals differ substantially from a healthy population, the disruption of the microbial community appears to be greater at a second time point later in the ICU stay, and a set of concerning inflammatory taxons co-occur. This study suggests that interventions focused on improving the microbiome in critical-care patients should be undertaken.

**FIG 1:**
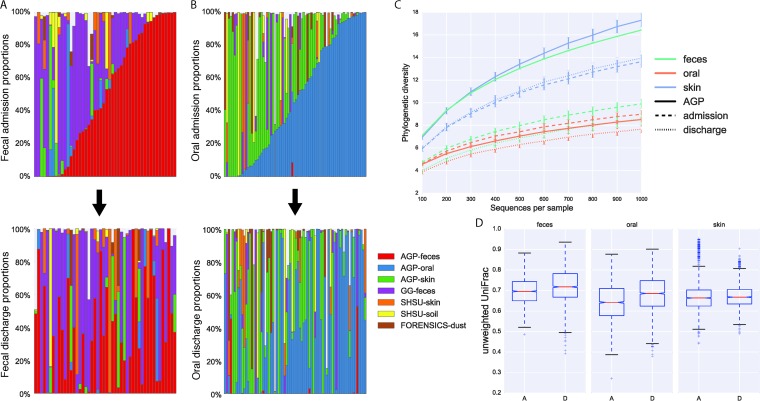
ICU stays result in drastic community changes. (A, B) SourceTracker proportions for ICU patients with samples obtained both at admission and at discharge in fecal (A) and oral (B) communities. The first row shows samples obtained at admission sorted by expected community type. The second shows samples obtained at discharge in patient order with the first row. Sources included samples from the healthy AGP subject subset, skin samples from decomposing bodies, soil samples from around decomposing bodies, fecal samples from healthy children in the Global Gut study, and dust samples from a house forensics study. A lack of color indicates an unknown source. For complete SourceTracker distribution plots, see Fig. S1 to 3 in the supplemental material. (C) Rarefaction curves using phylogenetic diversity of the ICU patient and healthy AGP subject samples. Error bars show standard errors. (D) Unweighted UniFrac distance distributions of within-time-point distances for fecal (*P* = 1.24e^−28^; Bonferroni corrected), oral (*P* = 1.75e^−71^; Bonferroni corrected), and skin (no significant difference) sites. The letters A and D on the *x* axis denote admission and discharge, respectively; whiskers are at 1.5 times the interquartile range. For a comparable weighted UniFrac analysis, see Fig. S4 in the supplemental material.

All ICU patient microbiome samples were assayed for community source proportions by using SourceTracker (11). Source samples were obtained from Qiita (https://qiita.ucsd.edu/) (14) and included samples from self-reportedly healthy individuals (15), healthy children (median age of 1.3 years) (16), dust from built-environment surfaces (17), and skin from decomposing human bodies and the soil surrounding the bodies (18). Source proportions for patients, where samples at both time points were viable, are shown in Fig. 1A and B for fecal and oral communities, respectively. Notably, at admission (first row in Fig. 1A and B), many of the samples appear to be of unexpected composition (e.g., an adult fecal sample resembling decomposing corpses). At discharge, a similar observation can be made, and the source proportions within an individual generally changed dramatically during this interval.

10.1128/mSphere.00199-16.5 Figure S1  SourceTracker distribution plots for all fecal samples. Download Figure S1, PDF file, 0.1 MB. Copyright © 2016 McDonald et al. 2016 McDonald et al. This content is distributed under the terms of the Creative Commons Attribution 4.0 International license.

10.1128/mSphere.00199-16.6 Figure S2  SourceTracker distribution plots for all oral samples. Download Figure S2, PDF file, 0.1 MB. Copyright © 2016 McDonald et al. 2016 McDonald et al. This content is distributed under the terms of the Creative Commons Attribution 4.0 International license.

10.1128/mSphere.00199-16.7 Figure S3  SourceTracker distribution proportions for all skin samples. Download Figure S3, PDF file, 0.1 MB. Copyright © 2016 McDonald et al. 2016 McDonald et al. This content is distributed under the terms of the Creative Commons Attribution 4.0 International license.

10.1128/mSphere.00199-16.8 Figure S4  Unweighted and weighted UniFrac distances by site partitioned by time point. Download Figure S4, PDF file, 0.1 MB. Copyright © 2016 McDonald et al. 2016 McDonald et al. This content is distributed under the terms of the Creative Commons Attribution 4.0 International license.

For fecal samples, the phylogenetic diversity (19) at discharge is significantly lower than at admission (nonparametric two-sample *t* test with 9,999 permutations, *P* = 0.045, Bonferroni corrected). Unweighted UniFrac (20) distance distributions of within-time-point distances significantly differ (Fig. 1D) by Kruskal-Wallis test for both fecal (*k* = 126.79, *P* = 1.24e^−28^, Bonferroni corrected) and oral communities (*k* = 323.2, *P* = 1.75e^−71^, Bonferroni corrected); however, a difference was not observed with skin, possibly because of the low-biomass nature of the samples and susceptibility to transient organisms (i.e., possible environmental normalization). Unexpectedly, fecal and oral samples obtained at admission are more similar to each other than to samples obtained at discharge, implying that the length of stay in an ICU is associated with community disruption.

**FIG 2:**
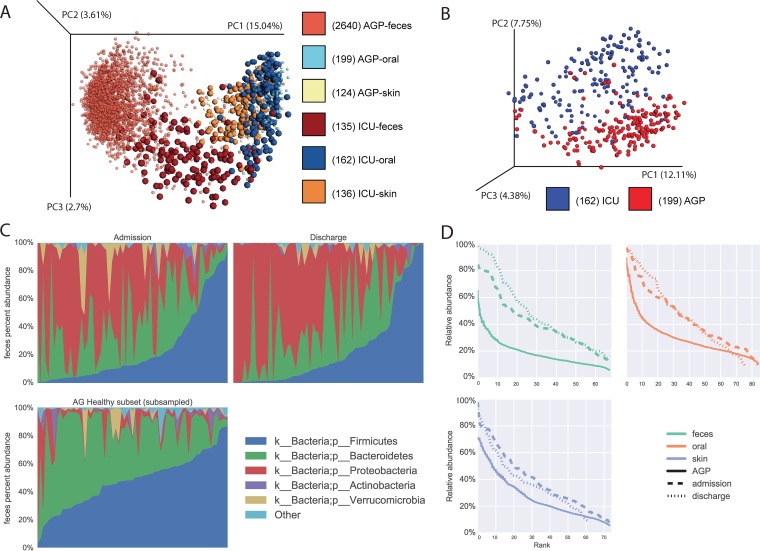
ICU patients differ markedly from healthy AGP subjects. (A, B) Principal-coordinate (PC) plots of unweighted UniFrac distances of both ICU patient and healthy AGP subject samples: A, fecal, skin, and oral samples with the ICU patient samples enlarged to aid in differentiation; B, oral samples in isolation. (C) Stacked taxonomy bar charts for fecal split by time point, showing a random subsample of healthy AGP subject samples. (D) Rank-abundance curves for all three body sites split by time point, showing random subsamples from the healthy AGP subject subset.

Principal-coordinate analysis of UniFrac distances of the ICU patient samples and oral samples in isolation relative to the healthy subset of the AGP samples (Fig. 2A and B) (15) shows strong separation. It is unlikely that this is a study effect, as the ICU patient samples were processed and run in parallel with AGP samples. These differences are further characterized by phylum-level taxonomy, where fecal samples from ICU patients tend to have a lower relative abundance of *Firmicutes* and *Bacteroidetes* and an increased relative abundance of *Proteobacteria* (Fig. 2C; see Fig. S5, oral and skin, in the supplemental material). We also observed large depletions of organisms previously thought to confer anti-inflammatory benefits, such as *Faecalibacterium* (21). Conversely, many of the taxa that increased contain well-recognized pathogens such as *Enterobacter* and *Staphylococcus* (see Tables S1 to S3 in the supplemental material). As previously observed in 14 ICU patients by Zaborin et al. (8), this disruption of the microbial community appears to generally associate with individual operational taxonomic units (OTUs) dominating a community, as observed at all of the body sites assayed (Fig. 2D). Interestingly, we observed inflammatory taxons appearing to co-occur when using SparCC (13) (co-occurrence analysis method as suggested in reference 22), including various members of the *Enterobacteriaceae* family, such as *Salmonella*, *Enterobacter*, *Citrobacter*, *Erwinia*, *Serratia*, and *Pantoea*. This observation was true at both time points and for both oral and fecal communities (see Fig. S6 in the supplemental material), suggesting a model of HAIs analogous to an unruly person looting vulnerable shops after a natural disaster, as unruly individuals tend to bring their unruly friends.

10.1128/mSphere.00199-16.2 Table S1  Significantly different OTUs assessed by ANCOM for each body site between the healthy AGP subject samples and ICU patient cohort samples. A significance level of 0.05 was used following multiple-hypothesis correction by the Holm-Bonferroni method. Download Table S1, XLSX file, 0.1 MB. Copyright © 2016 McDonald et al. 2016 McDonald et al. This content is distributed under the terms of the Creative Commons Attribution 4.0 International license.

10.1128/mSphere.00199-16.3 Table S2  ICU patient summary information for the 115 patients analyzed. Download Table S2, DOCX file, 0.1 MB. Copyright © 2016 McDonald et al. 2016 McDonald et al. This content is distributed under the terms of the Creative Commons Attribution 4.0 International license.

10.1128/mSphere.00199-16.4 Table S3  The samples used in the SourceTracker analysis. Download Table S3, XLSX file, 0.1 MB. Copyright © 2016 McDonald et al. 2016 McDonald et al. This content is distributed under the terms of the Creative Commons Attribution 4.0 International license.

10.1128/mSphere.00199-16.9 Figure S5  Stacked taxonomy plots for all samples partitioned by time point and sample type, as well as a random subset of AGP samples. Download Figure S5, PDF file, 0.3 MB. Copyright © 2016 McDonald et al. 2016 McDonald et al. This content is distributed under the terms of the Creative Commons Attribution 4.0 International license.

10.1128/mSphere.00199-16.10 Figure S6  Co-occurrence networks observed in fecal and oral communities suggesting that inflammatory clades co-occur. Blue edges indicate a positive correlation, and red indicates a negative correlation. The size of the edge shows the magnitude of the correlation. Layouts are the Cytoscape default. The labels used are the most informative taxon associated with the each OTU. All observed networks are available in the supplemental Cytoscape file. (A) Fecal samples obtained at admission. (B) Fecal samples obtained at discharge. (C) Oral samples obtained at admission. (D) Oral samples obtained at discharge. Download Figure S6, PDF file, 2.1 MB. Copyright © 2016 McDonald et al. 2016 McDonald et al. This content is distributed under the terms of the Creative Commons Attribution 4.0 International license.

In summary, this study tested the hypothesis that critical illness is associated with a loss of health-promoting commensal microbes and the occurrence of dysbiosis, which has previously been shown to be associated with high susceptibility to HAI, sepsis, and MODS (5, 6). Our analysis of microbiome data from 115 subjects indicates that the composition of the microbiome in many ICU patients is derived from unexpected sources and differs significantly from that of a healthy population and that the magnitude of this dysbiosis appears to increase between time points. Limitations of this study are described in the supplemental material; notably, the ubiquitous use of antibiotics precludes differentiating the effect of antibiotic pressure from the effect of critical illness. As >70% of ICU patients worldwide receive antibiotics (23), antibiotic pressure should be considered both a treatment and a fundamental pathophysiologic “insult” with likely negative effects on beneficial organisms. Irrespective of the cause, the evidence suggests that critical-care patients may benefit from therapeutics focused on improvement of the microbiome.

Previous research has shown that restoration of commensal “healthy microbes” following illness via interventions such as probiotics may exert their beneficial effects via multiple pathways, including suppression of dysbiosis or pathogenic microbes by inducing host cell antimicrobial peptides, release of antimicrobial factors, modulation of immune cell proliferation, stimulation of mucus and IgA production, inhibition of inflammatory epithelial cell nuclear factor kappa B activation, induction of mucin secretion, and other potentially vital gut epithelial barrier protective effects (24–26). As the gut is hypothesized to play a central role in the progression of critical illness, sepsis, and MODS (27), restoration of a healthy gut microbiome may be important for improving outcomes in critically ill patients. In support of this, a recent single case report of successful treatment of refractory severe sepsis and diarrhea with fecal transplantation has been published (28). Consistent with our findings, this ICU patient also demonstrated depleted *Firmicutes* and increased *Proteobacteria*, which was ameliorated by fecal transplantation, followed by rapid recovery of the patient (28).

In brief, methods for this study included fecal, oral, and skin sample collection by trained hospital personal from 115 mixed ICU patients >18 years of age who were mechanically ventilated within 48 h of ICU admission and were expected to remain in the ICU >72 h at five different ICUs. Patients were not excluded on the basis of health status. Samples were collected within 72 h of admission to the ICU and at discharge or on ICU day 10, where possible. Samples were then processed and sequenced by using Earth Microbiome Project (29) DNA protocols targeting the V4 region of the 16S rRNA gene (30) on the Illumina MiSeq platform. Sequence data were processed in QIIME 1.9.1 (31) by using Greengenes 13_8 (32). SourceTracker (11) was applied by using data sets sourced from Qiita (14). ANCOM (12) was applied to assess differential taxa between the ICU patient and AGP samples. For further details about the methods used, see Text S1 in the supplemental material.

10.1128/mSphere.00199-16.1 Text S1  Supplemental materials and methods, study design limitations, and supplemental references. Download Text S1, DOCX file, 0.05 MB. Copyright © 2016 McDonald et al. 2016 McDonald et al. This content is distributed under the terms of the Creative Commons Attribution 4.0 International license.

### Accession numbers.

The ICU patient sequence data and deidentified patient metadata are available in the Qiita database (https://qiita.ucsd.edu/) (accession no. 2136) and in the European Bioinformatics Institute, European Nucleotide Archive (accession no. ERP012810).

## References

[1] Human Microbiome Project Consortium 2012 Structure, function and diversity of the healthy human microbiome. Nature 486:207–214. doi:10.1038/nature11234. 22699609 PMC3564958

[2] Gevers D, Kugathasan S, Denson L, Vázquez-Baeza Y, Van Treuren W, Ren B, Schwager E, Knights D, Song S, Yassour M, Morgan X, Kostic A, Luo C, González A, McDonald D, Haberman Y, Walters T, Baker S, Rosh J, Stephens M, Heyman M, Markowitz J, Baldassano R, Griffiths A, Sylvester F, Mack D, Kim S, Crandall W, Hyams J, Huttenhower C, Knight R, Xavier RJ 2014 The treatment-naive microbiome in new-onset Crohn’s disease. Cell Host Microbe 15:382–392. doi:10.1016/j.chom.2014.02.005. 24629344 PMC4059512

[3] Weingarden A, González A, Vázquez-Baeza Y, Weiss S, Humphry G, Berg-Lyons D, Knights D, Unno T, Bobr A, Kang J, Khoruts A, Knight R, Sadowsky MJ 2015 Dynamic changes in short- and long-term bacterial composition following fecal microbiota transplantation for recurrent Clostridium difficile infection. Microbiome 3:10. doi:10.1186/s40168-015-0070-0. 25825673 PMC4378022

[4] Milbrandt EB, Kersten A, Rahim MT, Dremsizov TT, Clermont G, Cooper LM, Angus DC, Linde-Zwirble WT 2008 Growth of intensive care unit resource use and its estimated cost in Medicare. Crit Care Med 36:2504–2510. doi:10.1097/CCM.0b013e318183ef84. 18679127

[5] Alverdy JC, Chang EB 2008 The re-emerging role of the intestinal microflora in critical illness and inflammation: why the gut hypothesis of sepsis syndrome will not go away. J Leukoc Biol 83:461–466. doi:10.1189/jlb.0607372. 18160538

[6] Latorre M, Krishnareddy S, Freedberg D 2015 Microbiome as mediator: does systemic infection start in the gut? World J Gastroenterol 21:10487–10492. doi:10.3748/wjg.v21.i37.10487. 26457009 PMC4588071

[7] Klingensmith NJ, Coopersmith CM 2016 The gut as the motor of multiple organ dysfunction in critical illness. Crit Care Clin 32:203–212. doi:10.1016/j.ccc.2015.11.004. 27016162 PMC4808565

[8] Zaborin A, Smith D, Garfield K, Quensen J, Shakhsheer B, Kade M, Tirrell M, Tiedje J, Gilbert JA, Zaborina O, Alverdy JC 2014 Membership and behavior of ultra-low-diversity pathogen communities present in the gut of humans during prolonged critical illness. mBio 5:e01361-14. doi:10.1128/mBio.01361-14. 25249279 PMC4173762

[9] Ojima M, Motooka D, Shimizu K, Gotoh K, Shintani A, Yoshiya K, Nakamura S, Ogura H, Iida T, Shimazu T 2016 Metagenomic analysis reveals dynamic changes of whole gut microbiota in the acute phase of intensive care unit patients. Dig Dis Sci 61:1628–1634. doi:10.1007/s10620-015-4011-3. 26715502 PMC4875048

[10] Lyons JD, Ford ML, Coopersmith CM 2016 The microbiome in critical illness: firm conclusions or bact to square one? Dig Dis Sci 61:1420–1421. doi:10.1007/s10620-016-4092-7. 26923947 PMC4875832

[11] Knights D, Kuczynski J, Charlson ES, Zaneveld J, Mozer MC, Collman RG, Bushman FD, Knight R, Kelley ST 2011 Bayesian community-wide culture-independent microbial source tracking. Nat Methods 8:761–763. doi:10.1038/nmeth.1650. 21765408 PMC3791591

[12] Mandal S, Van Treuren W, White RA, Eggesbø M, Knight R, Peddada SD 2015 Analysis of composition of microbiomes: a novel method for studying microbial composition. Microb Ecol Health Dis 26:27663. doi:10.3402/mehd.v26.27663. 26028277 PMC4450248

[13] Friedman J, Alm EJ 2012 Inferring correlation networks from genomic survey data. PLoS Comput Biol 8:e1002687. doi:10.1371/journal.pcbi.1002687. 23028285 PMC3447976

[14] Qiita Development Team 2015 Qiita: spot patterns. https://pypi.python.org/pypi/qiita-spots.

[15] American Gut Project 2015 Human food project: anthropology of microbes. http://humanfoodproject.com/americangut/.

[16] Yatsunenko T, Rey FE, Manary MJ, Trehan I, Dominguez-Bello MG, Contreras M, Magris M, Hidalgo G, Baldassano RN, Anokhin AP, Heath AC, Warner B, Reeder J, Kuczynski J, Caporaso JG, Lozupone CA, Lauber C, Clemente JC, Knights D, Knight R, Gordon JI 2012 Human gut microbiome viewed across age and geography. Nature 486:222–227. doi:10.1038/nature11053. 22699611 PMC3376388

[17] Lax S, Smith DP, Hampton-Marcell J, Owens SM, Handley KM, Scott NM, Gibbons SM, Larsen P, Shogan BD, Weiss S, Metcalf JL, Ursell LK, Vazquez-Baeza Y, Van Treuren W, Hasan NA, Gibson MK, Colwell R, Dantas G, Knight R, Gilbert JA 2014 Longitudinal analysis of microbial interaction between humans and the indoor environment. Science 345:1048–1052. doi:10.1126/science.1254529. 25170151 PMC4337996

[18] Metcalf JL, Xu ZZ, Weiss S, Lax S, Van Treuren W, Hyde ER, Song SJ, Amir A, Larsen P, Sangwan N, Haarmann D, Humphrey GC, Ackermann G, Thompson LR, Lauber C, Bibat A, Nicholas C, Gebert MJ, Petrosino JF, Reed SC, Gilbert JA, Lynne AM, Bucheli SR, Carter DO, Knight R 2016 Microbial community assembly and metabolic function during mammalian corpse decomposition. Science 351:158–162. doi:10.1126/science.aad2646. 26657285

[19] Faith DP 1992 Conservation evaluation and phylogenetic diversity. Biol Conserv 61:1–10. doi:10.1016/0006-3207(92)91201-3.

[20] Lozupone C, Knight R 2005 UniFrac: a new phylogenetic method for comparing microbial communities. Appl Environ Microbiol 71:8228–8235. doi:10.1128/AEM.71.12.8228-8235.2005. 16332807 PMC1317376

[21] Sokol H, Pigneur B, Watterlot L, Lakhdari O, Bermudez-Humaran LG, Gratadoux JJ, Blugeon S, Bridonneau C, Furet JP, Corthier G, Grangette C, Vasquez N, Pochart P, Trugnan G, Thomas G, Blottiere HM, Dore J, Marteau P, Seksik P, Langella P 2008 Faecalibacterium prausnitzii is an anti-inflammatory commensal bacterium identified by gut microbiota analysis of Crohn disease patients. Proc Natl Acad Sci U S A 105:16731–16736. doi:10.1073/pnas.0804812105. 18936492 PMC2575488

[22] Weiss S, Van Treuren W, Lozupone C, Faust K, Friedman J, Deng Y, Xia LC, Xu ZZ, Ursell L, Alm EJ, Birmingham A, Cram JA, Fuhrman JA, Raes J, Sun F, Zhou J, Knight R 2016 Correlation detection strategies in microbial data sets vary widely in sensitivity and precision. ISME J 10:1669–1681. doi:10.1038/ismej.2015.235. 26905627 PMC4918442

[23] Vincent J, Marshall J, Anzueto A, Martin CD, Gomersall C 2009 International study of the prevalence and outcomes of infection in intensive care units. JAMA 302:2323–2329. doi:10.1001/jama.2009.1754. 19952319

[24] Luyer MD, Buurman WA, Hadfoune M, Speelmans G, Knol J, Jacobs JA, Dejong CHC, Vriesema AJM, Greve JWM 2005 Strain-specific effects of probiotics on gut barrier integrity following hemorrhagic shock. Infect Immun 73:3686–3692. doi:10.1128/IAI.73.6.3686-3692.2005. 15908398 PMC1111872

[25] Tok D, Ilkgul O, Bengmark S, Aydede H, Erhan Y, Taneli F, Ulman C, Vatansever S, Kose C, Ok G 2007 Pretreatment with pro- and synbiotics reduces peritonitis-induced acute lung injury in rats. J Trauma 62:880–885. doi:10.1097/01.ta.0000236019.00650.00. 17426542

[26] Corr SC, Li Y, Riedel CU, O’Toole PW, Hill C, Gahan CGM 2007 Bacteriocin production as a mechanism for the antiinfective activity of Lactobacillus salivarius UCC118. Proc Natl Acad Sci U S A 104:7617–7621. doi:10.1073/pnas.0700440104. 17456596 PMC1863472

[27] Mittal R, Coopersmith CM 2014 Redefining the gut as the motor of critical illness. Trends Mol Med 20:214–223. doi:10.1016/j.molmed.2013.08.004. 24055446 PMC3959633

[28] Li Q, Wang C, Tang C, He Q, Zhao X, Li N, Li J 2015 Successful treatment of severe sepsis and diarrhea after vagotomy utilizing fecal microbiota transplantation: a case report. Crit Care 19:37. doi:10.1186/s13054-015-0738-7. 25881250 PMC4346118

[29] Gilbert JA, Jansson JK, Knight R 2014 The Earth Microbiome Project: successes and aspirations. BMC Biol 12:69. doi:10.1186/s12915-014-0069-1. 25184604 PMC4141107

[30] Caporaso JG, Lauber CL, Walters WA, Berg-Lyons D, Huntley J, Fierer N, Owens SM, Betley J, Fraser L, Bauer M, Gormley N, Gilbert JA, Smith G, Knight R 2012 Ultra-high-throughput microbial community analysis on the Illumina HiSeq and MiSeq platforms. ISME J 6:1621–1624. doi:10.1038/ismej.2012.8. 22402401 PMC3400413

[31] Caporaso JG, Kuczynski J, Stombaugh J, Bittinger K, Bushman FD, Costello EK, Fierer N, Peña AG, Goodrich JK, Gordon JI, Huttley GA, Kelley ST, Knights D, Koenig JE, Ley RE, Lozupone CA, McDonald D, Muegge BD, Pirrung M, Reeder J, Sevinsky JR, Turnbaugh PJ, Walters WA, Widmann J, Yatsunenko T, Zaneveld J, Knight R 2010 QIIME allows analysis of high-throughput community sequencing data. Nat Methods 7:335–336. doi:10.1038/nmeth.f.303. 20383131 PMC3156573

[32] McDonald D, Price MN, Goodrich J, Nawrocki EP, DeSantis TZ, Probst A, Andersen GL, Knight R, Hugenholtz P 2012 An improved Greengenes taxonomy with explicit ranks for ecological and evolutionary analyses of bacteria and archaea. ISME J 6:610–618. doi:10.1038/ismej.2011.139. 22134646 PMC3280142

