# Supplementary figures and images for: Extreme Dysbiosis of the Microbiome in Critical Illness
Source: mSphere. 2016 Aug 31;1(4):e00199-16. doi: 10.1128/mSphere.00199-16 (PMC5007431; doi:10.1128/mSphere.00199-16)

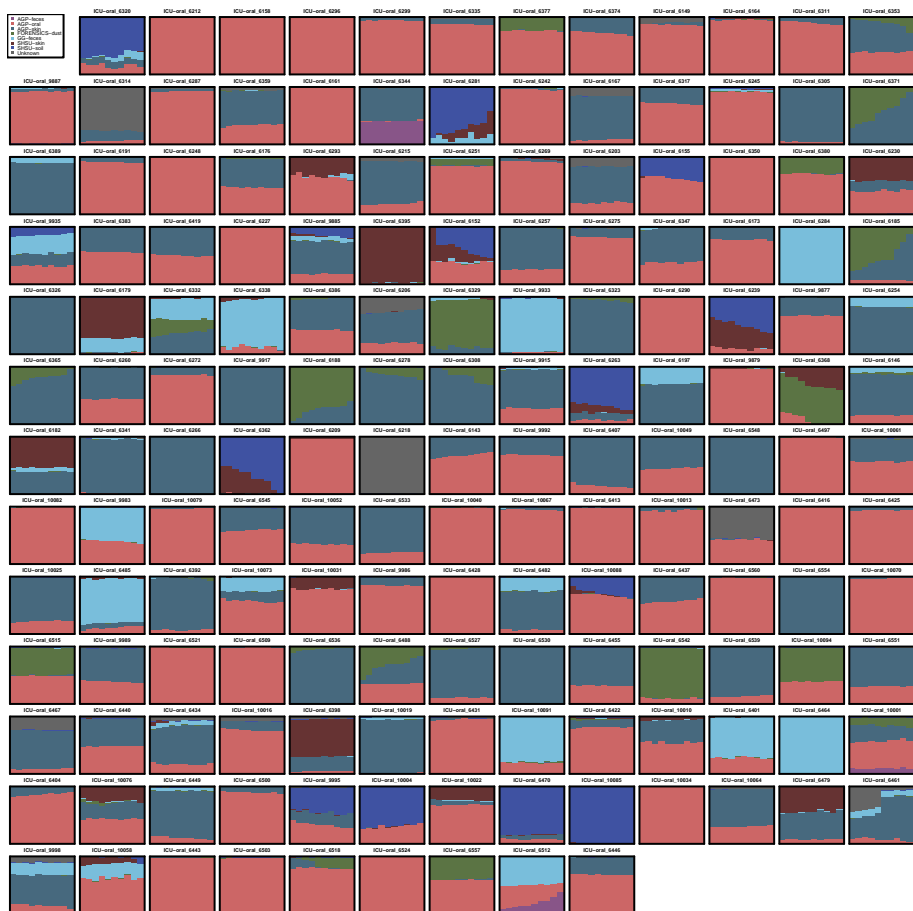

Supplement: Figure S2 [file sph004162139sf6.pdf]

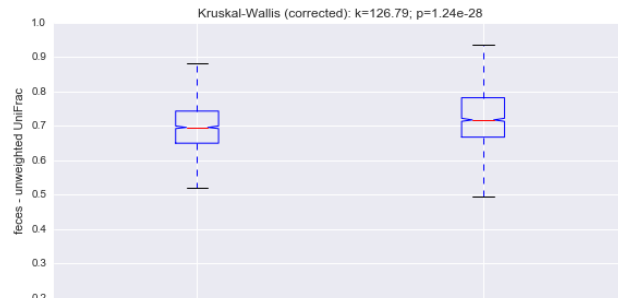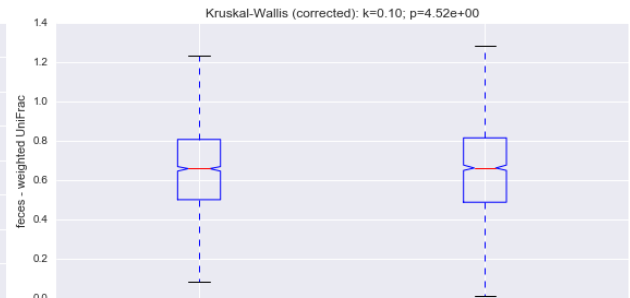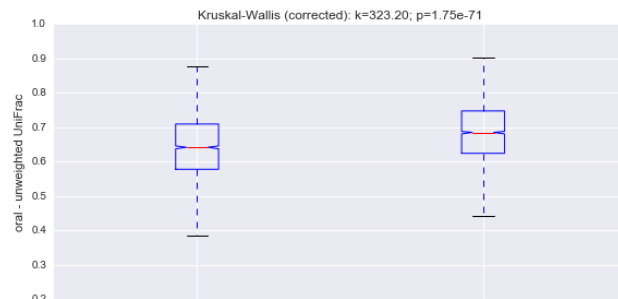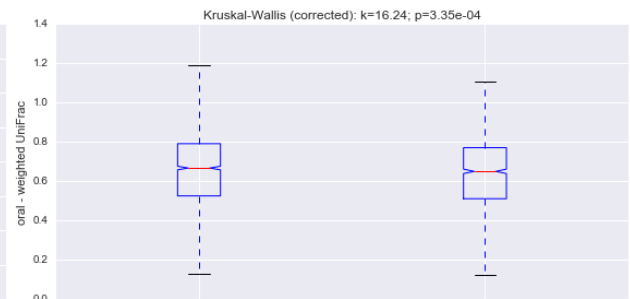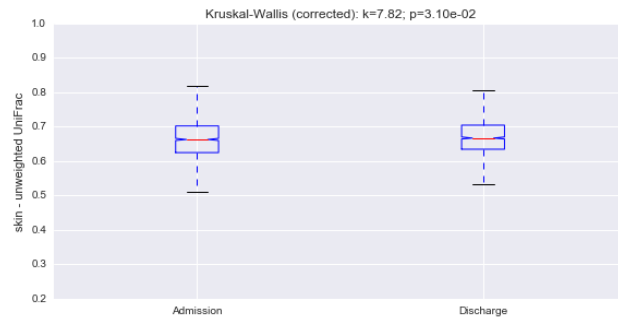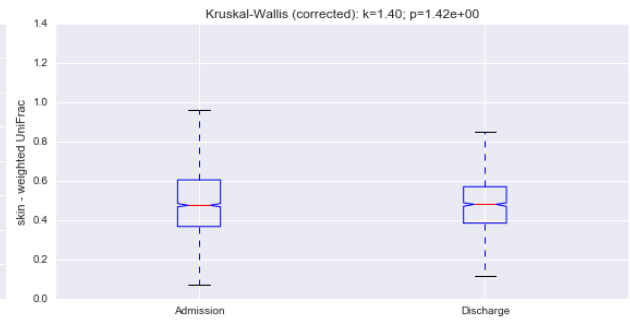

Supplement: Figure S4 [file sph004162139sf8.pdf]

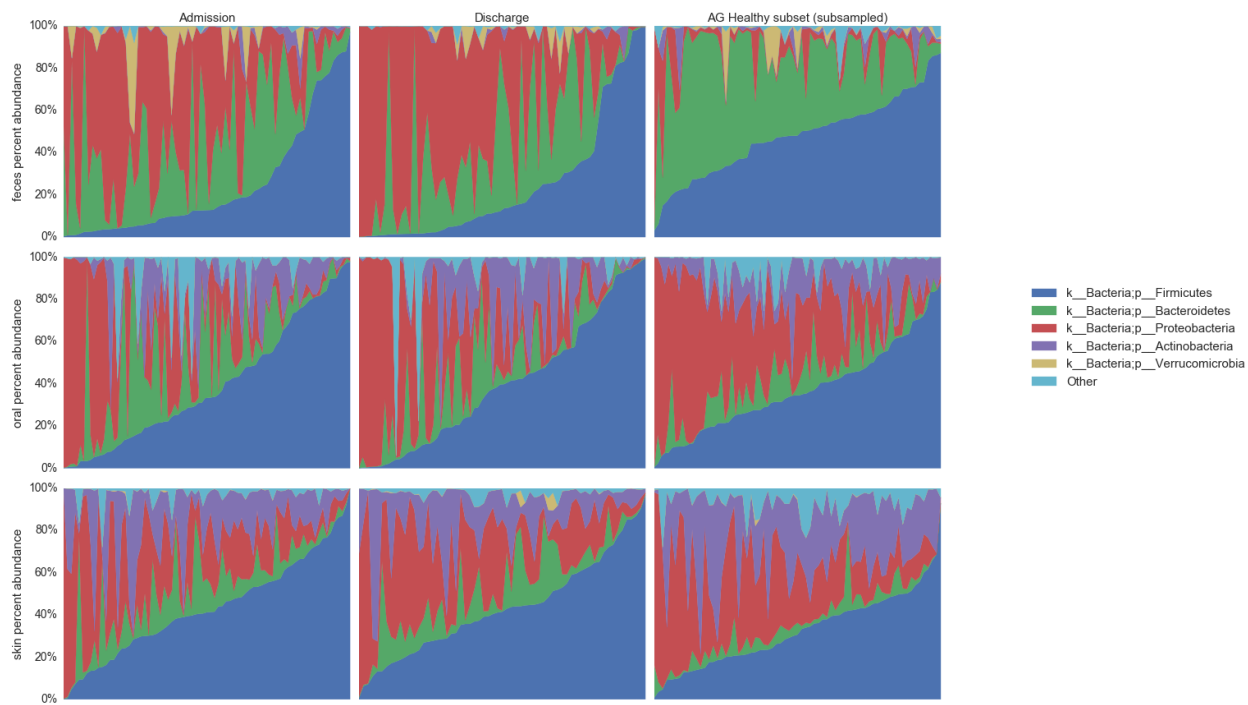

Supplement: Figure S5 [file sph004162139sf9.pdf]

A

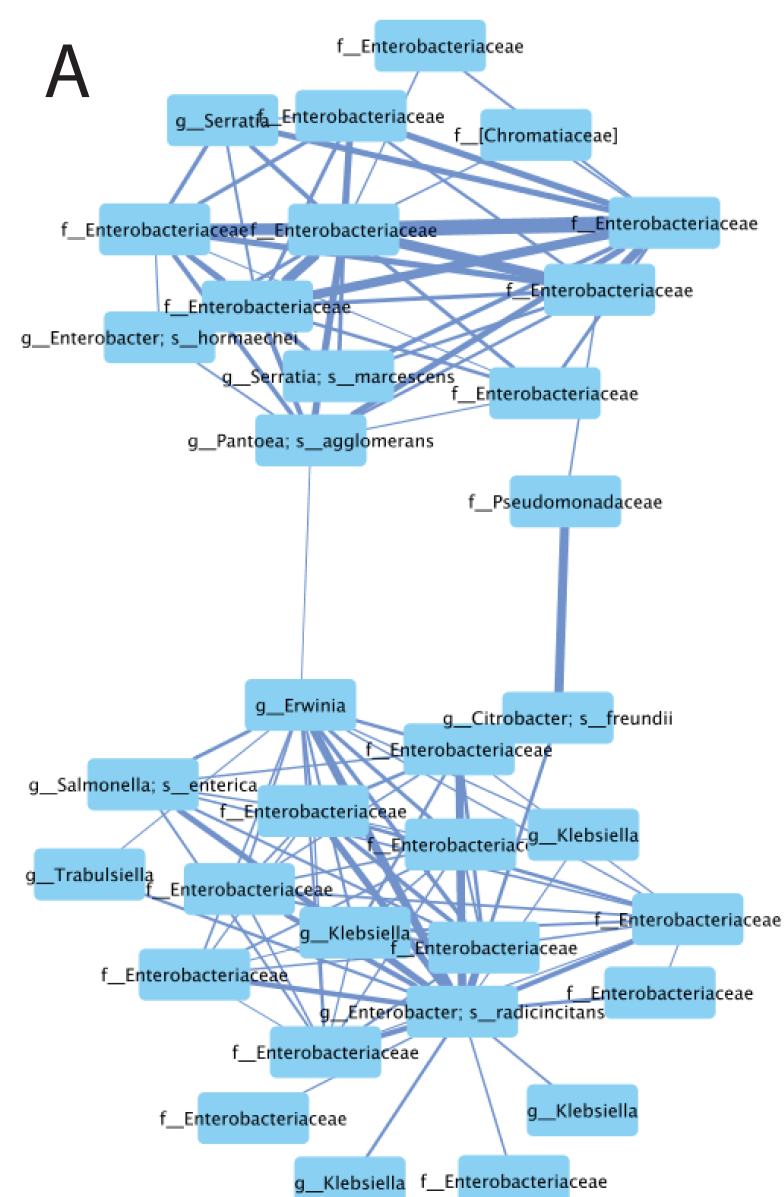

B

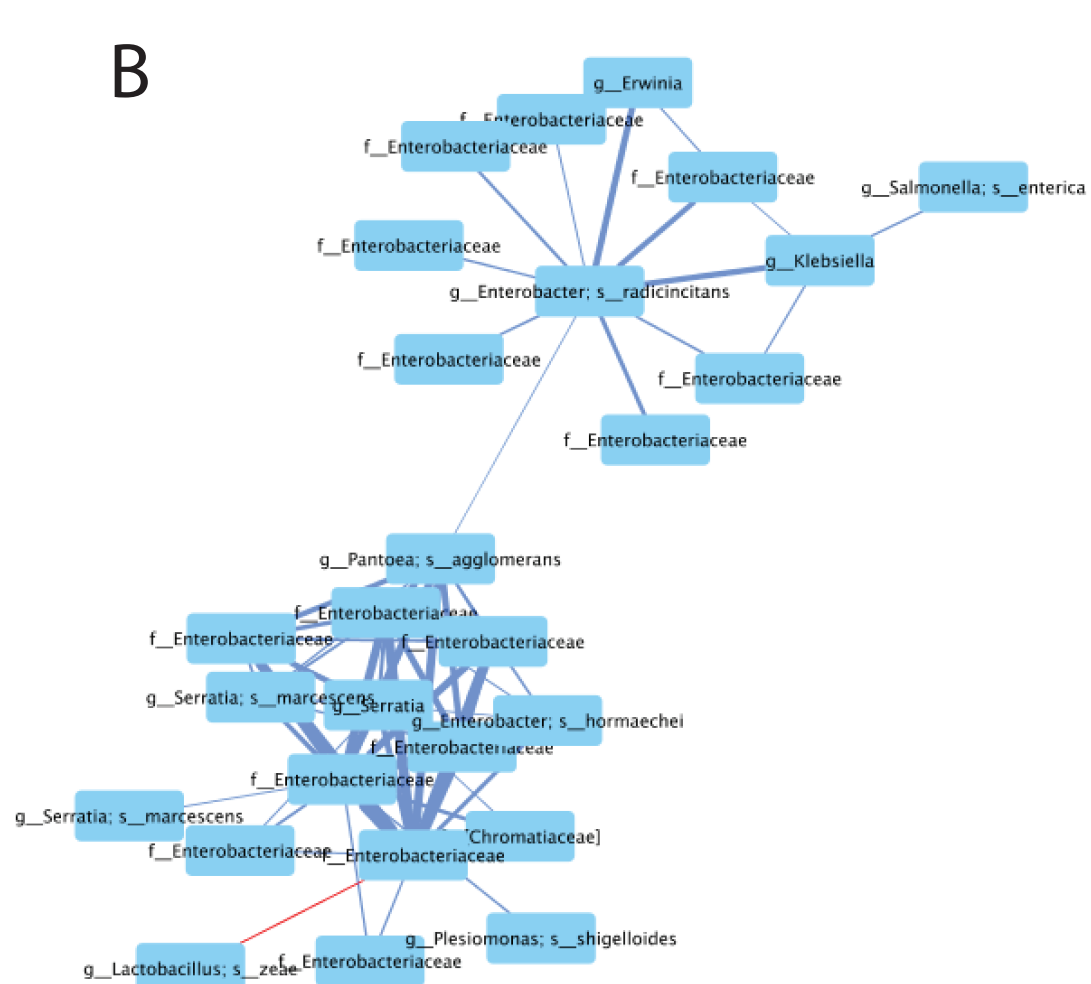

C

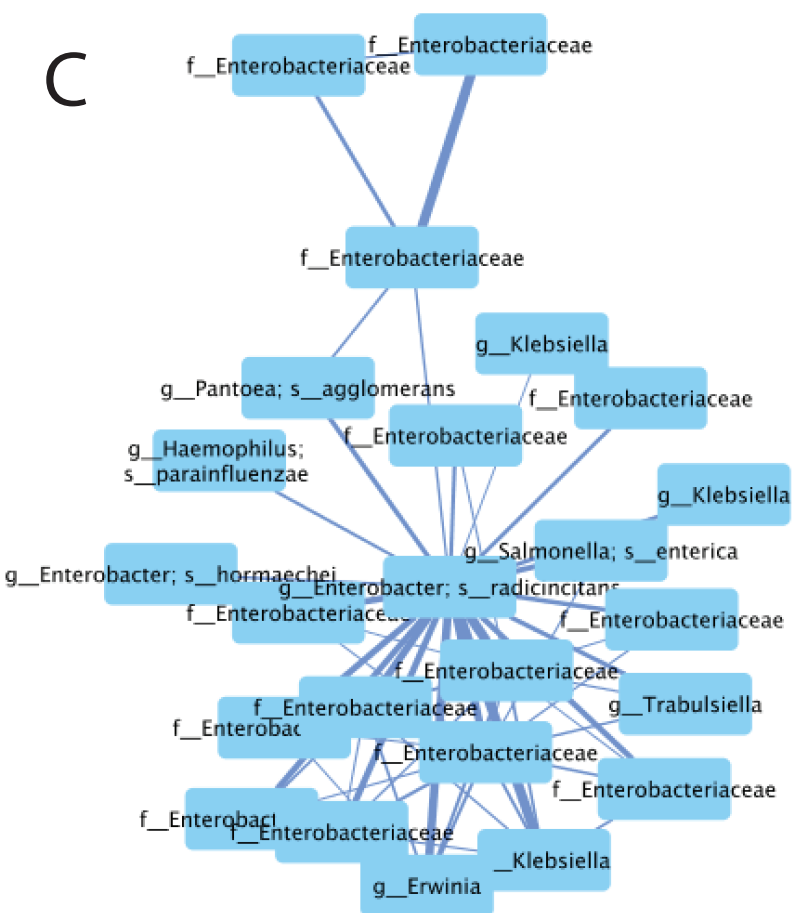

D

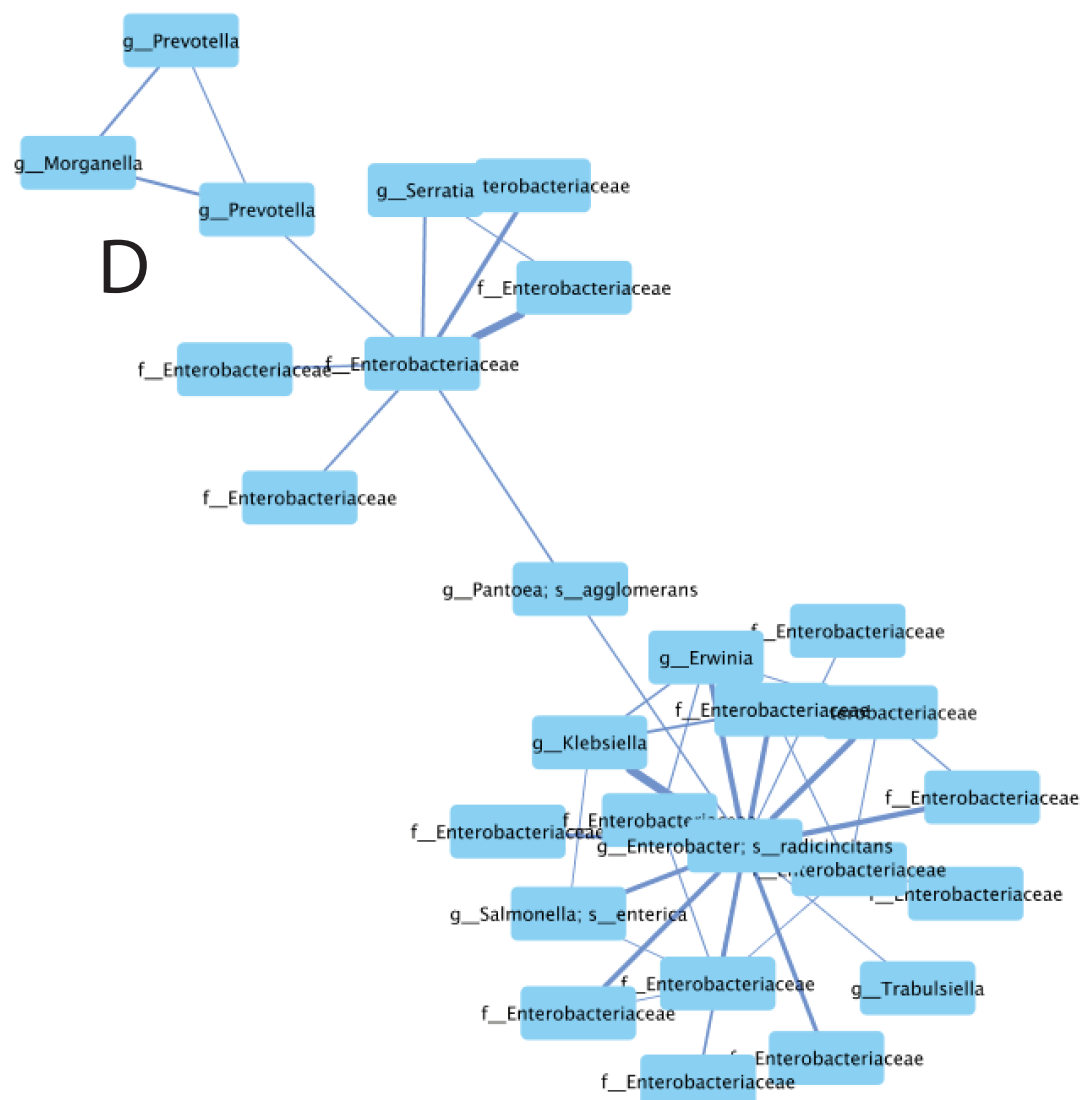

Supplement: Figure S6 [file sph004162139sf10.pdf]
