# Supplementary material for: Extreme Dysbiosis of the Microbiome in Critical Illness
Source: mSphere. 2016 Aug 31;1(4):e00199-16. doi: 10.1128/mSphere.00199-16 (PMC5007431; doi:10.1128/mSphere.00199-16)
Supplement: Table S2 [file sph004162139st3.docx]

**Table S2.** ICU patient summary information for the 115 patients analyzed.

| General |  |
| --- | --- |
| Age (y), mean (sd)  Males, n (%)  BMI (sd) | 54.5 (16.7)  63 (55%)  31.9 (11.1) |
| Illness Acuity  APACHE II, mean (sd)  Baseline SOFA (sd) | 23.7 (7.5)  8.2 (3.4) |
| Diagnosis  Medical (%)  Surgery/Trauma (%)  Surgical Emergent  Surgical Elective  Trauma  Medical Diagnosis Subtype  Sepsis  Neurologic  Respiratory  GI  Other  CV/Vascular  Metabolic | 60 (52%)  55 (48%)  39  17  8  8  18  17  5  9  2  1 |
| Clinical Outcomes  Antibiotics During ICU stay (%)  ICU Length of Stay (sd)  Hospital Length of Stay (sd)  Mechanical Ventilation Time (sd)  ARDS Diagnosis (%)  ICU Mortality (%)  Hospital Mortality (%) | 115 (100%)  17.3 (13.7)  25.0 (16.7)  11.1 (11.6)  10 (9%)  18 (15.7%)  24 (21%) |

Abbreviations: BMI- Body Mass Index, APACHE- Acute Physiology and Chronic Health Evaluation II Score, SOFA, Sequential Organ Failure Assessment Score, GI- Gastrointestinal, CV- Cardiovascular, ARDS- Acute Respiratory Distress Syndrome, ICU- Intensive Care Unit
