# Supplementary material for: Extreme Dysbiosis of the Microbiome in Critical Illness
Source: mSphere. 2016 Aug 31;1(4):e00199-16. doi: 10.1128/mSphere.00199-16 (PMC5007431; doi:10.1128/mSphere.00199-16)
Supplement: Text S1 [file sph004162139s1.docx]

**Supplemental**

**Materials and methods**

**Study design limitations**

**Supplemental references**

**Materials and Methods**

*Study population*

This project was reviewed and approved with a waiver of consent initially by the Colorado Multi-Institutional Review Board (COMIRB). This project was also reviewed and approved by all other participating sites ethics or IRB committees. When waiver of consent was granted at a site, all patients and families (when available) were provided with information sheets describing the study. If a patient or suitable proxy/ appointed decision maker indicated they did not want to participate in the study, any collected samples and data were destroyed and they were removed from study. Samples were collected from 115 mixed intensive care unit (ICU) patients >18 years of age who were mechanically ventilated within 48 hours of ICU admission and were expected to remain in the ICU > 72 hours at five different intensive care units (4 Hospital Centers in U.S. and Canada). Data was collected on ICU outcomes, including mortality. Patients were not excluded from the pilot based on health status. Fecal samples were collected within 72 hours of admission to the ICU, and at discharge or ICU day 10 where possible (median of 7 days between sample collections). A summary of the patient details can be found in table S2. The International Nutrition Survey coordinated by the Clinical Evaluation Research Unit, Kingston, Ontario was used as the platform to collect the clinical data.

*Sample collection*

Samples were collected by trained hospital clinical trial personnel using BBL Culture Swabs (Becton, Dickenson and Company; Sparks, Maryland). Samples were pre-allocated prior to delivery to the hospital staff, such that samples were grouped into per patient kits. Within a kit, each sample was consistently labeled to associate the sample with: the corresponding kit, the sample time point, and the specimen type. At or near the respective time points relative to a patient, oral and fecal samples were collected; skin was collected if the ICU was participating in that collection type. Fecal samples were collected by dipping the tips into defecated stool as close in time proximity as possible defecation. Oral swabs were collected by rubbing the swab tips on the surface of the patient's tongue; nurses were instructed to avoid lips and teeth. Skin samples, when collected, were from the foreheads of the patients; nurses were instructed to avoid hair.

*Sequencing and primary processing of sequence data*

Patient samples were processed and sequenced in parallel with American Gut samples using the standard Earth Microbiome Project (1) DNA protocols targeting the V4 region of the 16S rRNA gene (2). Samples were sequenced on an Illumina MiSeq by the sequencing core at the BioFrontiers Institute at the University of Colorado. The sequence data was then processed in QIIME 1.9.1 (3). Briefly, data were quality filtered and demultiplexed using default parameters. Forward reads were then trimmed to a maximum length of 100 nucleotides to reduce study effect in subsequent meta-analysis (see below). OTUs were picked against Greengenes 13_8 (4) at 97% sequence similarity using SortMeRNA 2.0 (5). This OTU table was then rarefied from 100 to 1000 sequences per sample in steps of 100 sequences at 10 iterations per step. Faith’s Phylogenetic Diversity (6) was then calculated using QIIME over the multiple rarefactions. Weighted and unweighted UniFrac (7) were calculated on a single table rarefied at 1000 sequences per sample.

*Aggregating data for SourceTracker*

Table S3 lists the specific subset of samples used as sources in SourceTracker. Briefly, data were obtained from Qiita (8) in February of 2016 and included the healthy American Gut subset samples (9) trimmed to 100nt, child samples from Yatsunenko et al. 2012 (10), skin and soil samples from Metcalf et al 2015 (11) decomposition study, and dust samples from Lax et al 2014 (12) house forensics study trimmed to 100nt. All combined studies were run on either the Illumina MiSeq or HiSeq platforms and processed using the Earth Microbiome Project (2) protocols, and all OTUs assayed were picked closed-reference against Greengenes 13_8 (4) at 97% sequence similarity using SortMeRNA 2.0 (5). The resulting OTU table was then randomly subsampled without replacement limit samples to a maximum of 50,000 sequences, and to drop samples with fewer than 100 reads in order to reduce a possible sequencing effort bias as the Yatsunenko et al 2012 (10) samples were sequenced to > 1 million reads per sample. A random set of samples were selected for each source such that each source contained the same number of samples (n=126 which was the minimum source size); this was done to avoid source size bias in SourceTracker. Any OTU observed in fewer than 10% of the samples was then dropped. SourceTracker 0.9.5 was then used with default parameters.

*Aggregating data for comparisons with the American Gut*

The healthy subset of the American Gut dataset was combined with the ICU samples. The resulting OTU table was rarefied to 1000 sequences per sample. Using QIIME (3), unweighted UniFrac was computed on this table, and then partitioned into two distance matrices: one which contains the three primary body sites, and one which contained only oral samples. Principal coordinates were then computed on both of these matrices, followed by visualization in EMPeror (13). Significantly different taxa by body site between American Gut healthy and the ICU samples were assessed using ANCOM (14) as implemented in scikit-bio (15) on 1000 sequence rarefied tables; OTUs represented in fewer than 10% of samples were dropped prior to rarefaction and calculation, and a significance level of 0.05 was used following multiple hypothesis correction using the Holm-Bonferroni method (16).

*Co-occurrence analysis*

The ICU samples were rarefied to 5,000 sequences per sample and partitioned by both body site and time point. OTUs present in < 10% of the samples within a given partition were removed. In accordance to the recommendations in (17), Inverse Simpson was calculated and the median effective species was found to be < 13 for each partition suggesting the use of SparCC (18) on the data. Each partition was run through SparCC (18) as implemented in PySurvey (19), using a correlation threshold of 0.35 (personal communication with Sophie Weiss). The resulting correlation network was visualized in Cytoscape (20).

*Figure generation*

Figures were generated either through a custom Jupyter Notebook (21) using matplotlib (22), Seaborn (23), pandas (24), scikit-bio (25), NumPy (26) and SciPy (26), with EMPeror (27), or with Cytoscape (20).

**Study design limitations**

Limitations of this study include the following. The study was conducted in a large number of ICU patients with varying diagnosis types and ages, although this is also a unique strength of the likely generalizability of the data. Some key limitations that contribute to increased variability in the data include the inability to control for the patients pre-ICU exposure to antibiotics, wide variations in antibiotic exposure (all patients in the study were treated with arbitrary and differing antibiotic regimens in the ICU), presence of concomitant chronic illness and potential pre-ICU prolonged hospitalization, varied ICU nutritional delivery, administration of acid suppression therapy, and as mentioned-diversity of age and ICU admission diagnosis. With regards to antibiotic pressure, Dethlefsen et al (28) observed inconsistent effects of antibiotics on the microbiome of healthy subjects. Thus, the dysbiosis and loss of diversity seen in ICU patients may not be entirely explained by antibiotic use. Further future subgroup analysis of this data and future targeted trials will help elucidate specific effects of these variables on the microbiome in specific ICU patient groups and should be pursued. The data of Zaborin et al (29) is an excellent example of a targeted ICU microbiome analysis in 4 patients with prolonged length of stay, selected from a 14 patient ICU cohort, but covers far fewer subjects.

**Supplemental references**

1. **Gilbert JA**, **Jansson JK**, **Knight R**. 2014. The Earth Microbiome project: successes and aspirations. BMC Biol **12**:69.

2. **Caporaso JG**, **Lauber CL**, **Walters WA**, **Berg-Lyons D**, **Huntley J**, **Fierer N**, **Owens SM**, **Betley J**, **Fraser L**, **Bauer M**, **Gormley N**, **Gilbert JA**, **Smith G**, **Knight R**. 2012. Ultra-high-throughput microbial community analysis on the Illumina HiSeq and MiSeq platforms. ISME J **6**:1621–1624.

3. **Caporaso JG**, **Kuczynski J**, **Stombaugh J**, **Bittinger K**, **Bushman FD**, **Costello EK**, **Fierer N**, **Pena AG**, **Goodrich JK**, **Gordon JI**, **Huttley GA**, **Kelley ST**, **Knights D**, **Koenig JE**, **Ley RE**, **Lozupone CA**, **McDonald D**, **Muegge BD**, **Pirrung M**, **Reeder J**, **Sevinsky JR**, **Turnbaugh PJ**, **Walters WA**, **Widmann J**, **Yatsunenko T**, **Zaneveld J**, **Knight R**. 2010. QIIME allows analysis of high-throughput community sequencing data. Nat Methods **7**:335–336.

4. **McDonald D**, **Price MN**, **Goodrich J**, **Nawrocki EP**, **DeSantis TZ**, **Probst A**, **Andersen GL**, **Knight R**, **Hugenholtz P**. 2012. An improved Greengenes taxonomy with explicit ranks for ecological and evolutionary analyses of bacteria and archaea. ISME J **6**:610–618.

5. **Kopylova E**, **Noe L**, **Touzet H**. 2012. SortMeRNA: fast and accurate filtering of ribosomal RNAs in metatranscriptomic data. Bioinformatics **28**:3211–3217.

6. **Faith DP**. 1992. Conservation evaluation and phylogenetic diversity. Biol Conserv **61**:1–10.

7. **Lozupone C**, **Knight R**. 2005. UniFrac: a new phylogenetic method for comparing microbial communities. Appl Env Microbiol **71**:8228–8235.

8. **Qiita-development-team**. Qiita: spot patterns.

9. **American-Gut-Project**. 2015. Website.

10. **Yatsunenko T**, **Rey FE**, **Manary MJ**, **Trehan I**, **Dominguez-Bello MG**, **Contreras M**, **Magris M**, **Hidalgo G**, **Baldassano RN**, **Anokhin AP**, **Heath AC**, **Warner B**, **Reeder J**, **Kuczynski J**, **Caporaso JG**, **Lozupone CA**, **Lauber C**, **Clemente JC**, **Knights D**, **Knight R**, **Gordon JI**. 2012. Human gut microbiome viewed across age and geography. Nature **486**:222–227.

11. **Metcalf JL**, **Xu ZZ**, **Weiss S**, **Lax S**, **Treuren W Van**, **Hyde ER**, **Song SJ**, **Amir A**, **Larsen P**, **Sangwan N**, **Haarmann D**, **Humphrey GC**, **Ackermann G**, **Thompson LR**, **Lauber C**, **Bibat A**, **Nicholas C**, **Gebert MJ**, **Petrosino JF**, **Reed SC**, **Gilbert JA**, **Lynne AM**, **Bucheli SR**, **Carter DO**, **Knight R**. 2016. Microbial community assembly and metabolic function during mammalian corpse decomposition. Science (80- ) **351**:158–162.

12. **Lax S**, **Smith DP**, **Hampton-Marcell J**, **Owens SM**, **Handley KM**, **Scott NM**, **Gibbons SM**, **Larsen P**, **Shogan BD**, **Weiss S**, **Metcalf JL**, **Ursell LK**, **Vazquez-Baeza Y**, **Van Treuren W**, **Hasan NA**, **Gibson MK**, **Colwell R**, **Dantas G**, **Knight R**, **Gilbert JA**. 2014. Longitudinal analysis of microbial interaction between humans and the indoor environment. Science (80- ) **345**:1048–1052.

13. **Vázquez-Baeza Y**, **Pirrung M**, **Gonzalez A**, **Knight R**. 2013. EMPeror: a tool for visualizing high-throughput microbial community data. Gigascience **2**:16.

14. **Mandal S**, **Van Treuren W**, **White RA**, **Eggesbø M**, **Knight R**, **Peddada SD**. 2015. Analysis of composition of microbiomes: a novel method for studying microbial composition. Microb Ecol Health Dis **26**:27663.

15. **Rideout JR**, **Gonzalez A**, **Treuren W Van**, **Morton J**, **Luce L**, **adamrp**, **Shorenstein J**, **Navas J**, **Pitman A**, **jradinger**, **Patena W**, **shiffer1**, **Debelius JW**, **Baeza YV**, **Brislawn C**, **charudatta-navare**, **alexbrc**, **nbresnick**, **McDonald D**, **Gorlick K**, **Caporaso G**, **Xu Z**, **Chase J**, **Bolyen E**, **Reeder J**, **Schwarzberg K**, **teravest**, **Murray K**, **Alastuey JC**, **Cope C**. 2016. scikit-bio: scikit-bio 0.4.2: Maintenance release.

16. **Holm S**. 1979. A Simple Sequentially Rejective Multiple Test Procedure. Scand J Stat **6**:65–70.

17. **Weiss S**, **Van Treuren W**, **Lozupone C**, **Faust K**, **Friedman J**, **Deng Y**, **Xia LC**, **Xu ZZ**, **Ursell L**, **Alm EJ**, **Birmingham A**, **Cram J a**, **Fuhrman J a**, **Raes J**, **Sun F**, **Zhou J**, **Knight R**. 2016. Correlation detection strategies in microbial data sets vary widely in sensitivity and precision. Isme J 1–13.

18. **Friedman J**, **Alm EJ**. 2012. Inferring Correlation Networks from Genomic Survey Data. PLoS Comput Biol **8**.

19. **Friedman J**. 2013. PySurvey is a Python package designed to perform interactive analysis of survey data, composed of counts of occurrence of different categories in a collection of samples.

20. **Cline MS**, **Smoot M**, **Cerami E**, **Kuchinsky A**, **Landys N**, **Workman C**, **Christmas R**, **Avila-Campilo I**, **Creech M**, **Gross B**, **Hanspers K**, **Isserlin R**, **Kelley R**, **Killcoyne S**, **Lotia S**, **Maere S**, **Morris J**, **Ono K**, **Pavlovic V**, **Pico AR**, **Vailaya A**, **Wang PL**, **Adler A**, **Conklin BR**, **Hood L**, **Kuiper M**, **Sander C**, **Schmulevich I**, **Schwikowski B**, **Warner GJ**, **Ideker T**, **Bader GD**. 2007. Integration of biological networks and gene expression data using Cytoscape. Nat Protoc **2**:2366–2382.

21. **F Perez BRG**. 2007. IPython: a system for interactive scientific computing. Comput Sci Eng **9**:21–29.

22. **Hunter JD**. 2007. Matplotlib: A 2D graphics environment. Comput Sci Eng **9**:99–104.

23. **Waskom M**, **St-Jean S**, **Evans C**, **Warmenhoven J**, **Meyer K**, **Martin M**, **Rocher L**, **Hobson P**, **Bachant P**, **Nagy T**, **Wehner D**, **Botvinnik O**, **Megies T**, **Lukauskas S**, **drewokane**, **Ziegler E**, **Yarkoni T**, **Miles A**, **Lee A**, **Coelho LP**, **Halchenko Y**, **Augspurger T**, **Hitz G**, **Vanderplas J**, **Fitzgerald C**, **Cole JB**, **gkunter**, **Villalba S**, **Hoyer S**, **Quintero E**. 2016. seaborn: v0.7.0 (January 2016).

24. **McKinney W**. 2010. Data Structures for Statistical Computing in Python, p. 51–56. *In* van der Walt, S, Millman, J (eds.), Proceedings of the 9th Python in Science Conference.

25. **Caporaso JG**. 2015. scikit-bio.

26. **Van Der Walt S**, **Colbert SC**, **Varoquaux G**. 2011. The NumPy array: A structure for efficient numerical computation. Comput Sci Eng **13**:22–30.

27. **Vazquez-Baeza Y**, **Pirrung M**, **Gonzalez A**, **Knight R**. 2013. EMPeror: a tool for visualizing high-throughput microbial community data. Gigascience **2**:16.

28. **Dethlefsen L**, **Huse S**, **Sogin ML**, **Relman DA**. 2008. The pervasive effects of an antibiotic on the human gut microbiota, as revealed by deep 16S rRNA sequencing. PLoS Biol **6**:e280.

29. **Zaborin A**, **Smith D**, **Garfield K**, **Quensen J**, **Shakhsheer B**, **Kade M**, **Tirrell M**, **Tiedje J**, **Gilbert JA**, **Zaborina O**, **Alverdy JC**. 2014. Membership and behavior of ultra-low-diversity pathogen communities present in the gut of humans during prolonged critical illness. MBio **5**.
