# Supplementary material for: Extreme Dysbiosis of the Microbiome in Critical Illness
Source: mSphere. 2016 Aug 31;1(4):e00199-16. doi: 10.1128/mSphere.00199-16 (PMC5007431; doi:10.1128/mSphere.00199-16)
Supplement: Figure S1 [file sph004162139sf5.pdf]

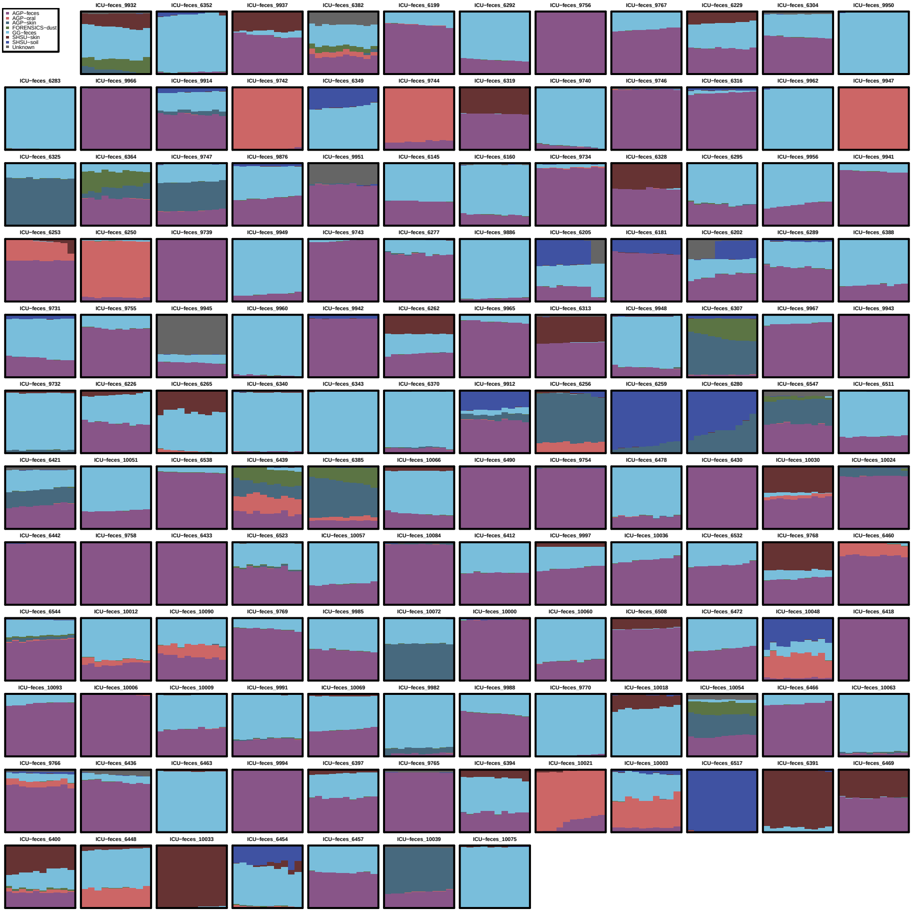

AGP−feces
AGP−oral
AGP−skin
FORENSICS−dust
GG−feces
SHSU−skin
SHSU−soil
Unknown
ICU−feces_9932
ICU−feces_6352
ICU−feces_9937
ICU−feces_6382
ICU−feces_6199
ICU−feces_6292
ICU−feces_9756
ICU−feces_9767
ICU−feces_6229
ICU−feces_6304
ICU−feces_9950
ICU−feces_6283
ICU−feces_9966
ICU−feces_9914
ICU−feces_9742
ICU−feces_6349
ICU−feces_9744
ICU−feces_6319
ICU−feces_9740
ICU−feces_9746
ICU−feces_6316
ICU−feces_9962
ICU−feces_9947
ICU−feces_6325
ICU−feces_6364
ICU−feces_9747
ICU−feces_9876
ICU−feces_9951
ICU−feces_6145
ICU−feces_6160
ICU−feces_9734
ICU−feces_6328
ICU−feces_6295
ICU−feces_9956
ICU−feces_9941
ICU−feces_6253
ICU−feces_6250
ICU−feces_9739
ICU−feces_9949
ICU−feces_9743
ICU−feces_6277
ICU−feces_9886
ICU−feces_6205
ICU−feces_6181
ICU−feces_6202
ICU−feces_6289
ICU−feces_6388
ICU−feces_9731
ICU−feces_9755
ICU−feces_9945
ICU−feces_9960
ICU−feces_9942
ICU−feces_6262
ICU−feces_9965
ICU−feces_6313
ICU−feces_9948
ICU−feces_6307
ICU−feces_9967
ICU−feces_9943
ICU−feces_9732
ICU−feces_6226
ICU−feces_6265
ICU−feces_6340
ICU−feces_6343
ICU−feces_6370
ICU−feces_9912
ICU−feces_6256
ICU−feces_6259
ICU−feces_6280
ICU−feces_6547
ICU−feces_6511
ICU−feces_6421
ICU−feces_10051
ICU−feces_6538
ICU−feces_6439
ICU−feces_6385
ICU−feces_10066
ICU−feces_6490
ICU−feces_9754
ICU−feces_6478
ICU−feces_6430
ICU−feces_10030
ICU−feces_10024
ICU−feces_6442
ICU−feces_9758
ICU−feces_6433
ICU−feces_6523
ICU−feces_10057
ICU−feces_10084
ICU−feces_6412
ICU−feces_9997
ICU−feces_10036
ICU−feces_6532
ICU−feces_9768
ICU−feces_6460
ICU−feces_6544
ICU−feces_10012
ICU−feces_10090
ICU−feces_9769
ICU−feces_9985
ICU−feces_10072
ICU−feces_10000
ICU−feces_10060
ICU−feces_6508
ICU−feces_6472
ICU−feces_10048
ICU−feces_6418
ICU−feces_10093
ICU−feces_10006
ICU−feces_10009
ICU−feces_9991
ICU−feces_10069
ICU−feces_9982
ICU−feces_9988
ICU−feces_9770
ICU−feces_10018
ICU−feces_10054
ICU−feces_6466
ICU−feces_10063
ICU−feces_9766
ICU−feces_6436
ICU−feces_6463
ICU−feces_9994
ICU−feces_6397
ICU−feces_9765
ICU−feces_6394
ICU−feces_10021
ICU−feces_10003
ICU−feces_6517
ICU−feces_6391
ICU−feces_6469
ICU−feces_6400
ICU−feces_6448
ICU−feces_10033
ICU−feces_6454
ICU−feces_6457
ICU−feces_10039
ICU−feces_10075
