# Supplementary material for: Extreme Dysbiosis of the Microbiome in Critical Illness
Source: mSphere. 2016 Aug 31;1(4):e00199-16. doi: 10.1128/mSphere.00199-16 (PMC5007431; doi:10.1128/mSphere.00199-16)
Supplement: Figure S3 [file sph004162139sf7.pdf]

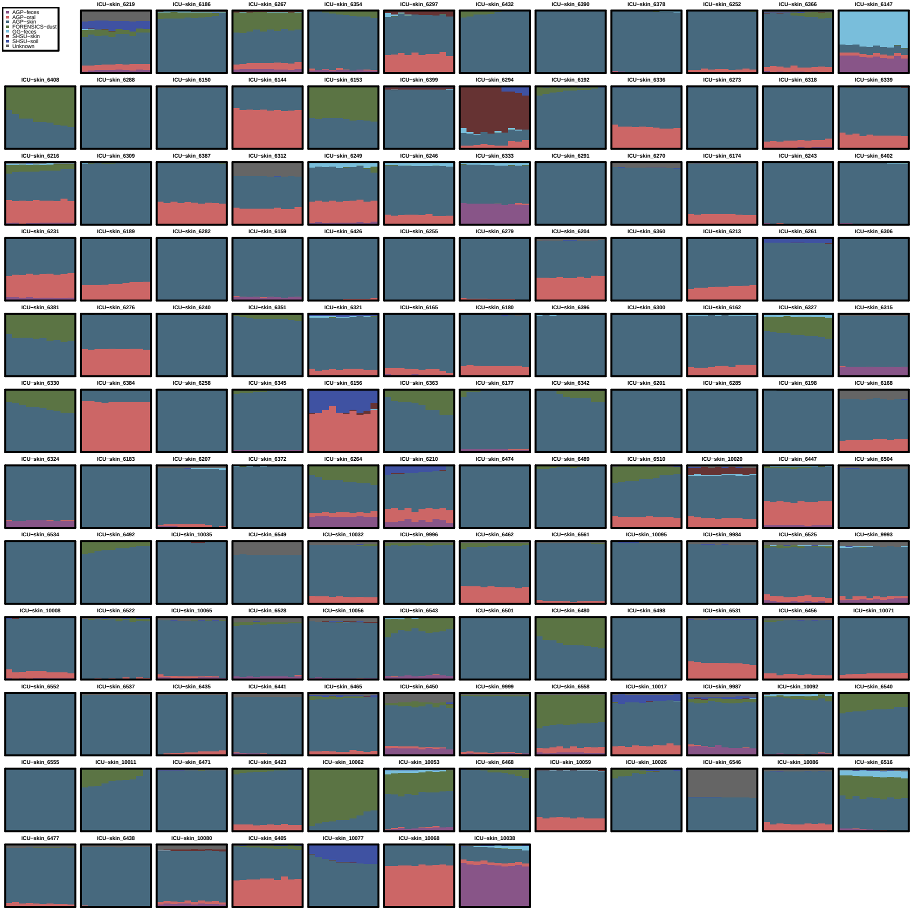
AGP−feces
AGP−oral
AGP−skin
FORENSICS−dust
GG−feces
SHSU−skin
SHSU−soil
Unknown
ICU−skin_6219
ICU−skin_6186
ICU−skin_6267
ICU−skin_6354
ICU−skin_6297
ICU−skin_6432
ICU−skin_6390
ICU−skin_6378
ICU−skin_6252
ICU−skin_6366
ICU−skin_6147
ICU−skin_6408
ICU−skin_6288
ICU−skin_6150
ICU−skin_6144
ICU−skin_6153
ICU−skin_6399
ICU−skin_6294
ICU−skin_6192
ICU−skin_6336
ICU−skin_6273
ICU−skin_6318
ICU−skin_6339
ICU−skin_6216
ICU−skin_6309
ICU−skin_6387
ICU−skin_6312
ICU−skin_6249
ICU−skin_6246
ICU−skin_6333
ICU−skin_6291
ICU−skin_6270
ICU−skin_6174
ICU−skin_6243
ICU−skin_6402
ICU−skin_6231
ICU−skin_6189
ICU−skin_6282
ICU−skin_6159
ICU−skin_6426
ICU−skin_6255
ICU−skin_6279
ICU−skin_6204
ICU−skin_6360
ICU−skin_6213
ICU−skin_6261
ICU−skin_6306
ICU−skin_6381
ICU−skin_6276
ICU−skin_6240
ICU−skin_6351
ICU−skin_6321
ICU−skin_6165
ICU−skin_6180
ICU−skin_6396
ICU−skin_6300
ICU−skin_6162
ICU−skin_6327
ICU−skin_6315
ICU−skin_6330
ICU−skin_6384
ICU−skin_6258
ICU−skin_6345
ICU−skin_6156
ICU−skin_6363
ICU−skin_6177
ICU−skin_6342
ICU−skin_6201
ICU−skin_6285
ICU−skin_6198
ICU−skin_6168
ICU−skin_6324
ICU−skin_6183
ICU−skin_6207
ICU−skin_6372
ICU−skin_6264
ICU−skin_6210
ICU−skin_6474
ICU−skin_6489
ICU−skin_6510
ICU−skin_10020
ICU−skin_6447
ICU−skin_6504
ICU−skin_6534
ICU−skin_6492
ICU−skin_10035
ICU−skin_6549
ICU−skin_10032
ICU−skin_9996
ICU−skin_6462
ICU−skin_6561
ICU−skin_10095
ICU−skin_9984
ICU−skin_6525
ICU−skin_9993
ICU−skin_10008
ICU−skin_6522
ICU−skin_10065
ICU−skin_6528
ICU−skin_10056
ICU−skin_6543
ICU−skin_6501
ICU−skin_6480
ICU−skin_6498
ICU−skin_6531
ICU−skin_6456
ICU−skin_10071
ICU−skin_6552
ICU−skin_6537
ICU−skin_6435
ICU−skin_6441
ICU−skin_6465
ICU−skin_6450
ICU−skin_9999
ICU−skin_6558
ICU−skin_10017
ICU−skin_9987
ICU−skin_10092
ICU−skin_6540
ICU−skin_6555
ICU−skin_10011
ICU−skin_6471
ICU−skin_6423
ICU−skin_10062
ICU−skin_10053
ICU−skin_6468
ICU−skin_10059
ICU−skin_10026
ICU−skin_6546
ICU−skin_10086
ICU−skin_6516
ICU−skin_6477
ICU−skin_6438
ICU−skin_10080
ICU−skin_6405
ICU−skin_10077
ICU−skin_10068
ICU−skin_10038
